# Exploring postictal recovery with acetaminophen or nimodipine: A randomized‐controlled crossover trial

**DOI:** 10.1002/acn3.52143

**Published:** 2024-08-19

**Authors:** Julia C. M. Pottkämper, Joey P. A. J. Verdijk, Sven Stuiver, Eva Aalbregt, Freek ten Doesschate, Esmée Verwijk, Martin Schmettow, Guido A. van Wingen, Michel J. A. M. van Putten, Jeannette Hofmeijer, Jeroen A. van Waarde

**Affiliations:** ^1^ Clinical Neurophysiology group University of Twente Enschede The Netherlands; ^2^ Department of Psychiatry Rijnstate Hospital P.O. Box 9555 Arnhem 6800 TA The Netherlands; ^3^ Department of Neurology Rijnstate Hospital P.O. Box 9555 Arnhem 6800 TA The Netherlands; ^4^ Department of Psychiatry Amsterdam UMC location University of Amsterdam Amsterdam The Netherlands; ^5^ Department of Radiology and Nuclear Medicine Amsterdam UMC location University of Amsterdam P.O. Box 22660 Amsterdam 1100 DD The Netherlands; ^6^ Department of Medical Psychology Amsterdam UMC location University of Amsterdam P.O. Box 22660 Amsterdam 1100 DD The Netherlands; ^7^ Department of Psychology Amsterdam University P.O. Box 19268 Amsterdam 1000 GG The Netherlands; ^8^ Department of Cognitive Psychology and Ergonomics, Faculty of Behavioural, Management and Social Sciences University of Twente P.O. Box 217 Enschede 7500 AE the Netherlands; ^9^ Department of Neurology and Clinical Neurophysiology Medisch spectrum Twente P.O. Box 50000 Enschede 7500 KA The Netherlands

## Abstract

**Objective:**

The postictal state is underrecognized in epilepsy. Animal models show improvement of postictal symptoms and cerebral perfusion with acetaminophen or nimodipine. We studied the effects of acetaminophen or nimodipine on postictal electroencephalographic (EEG) recovery, clinical reorientation, and hypoperfusion in patients with ECT‐induced seizures.

**Methods:**

In this prospective clinical trial with three‐condition randomized crossover design, study interventions were administered orally 2 h before ECT sessions (1000 mg acetaminophen, 60 mg nimodipine, or a placebo condition). Primary outcome measure was the speed of postictal EEG recovery. Secondary outcomes were the extent of postictal EEG recovery, clinical reorientation time, and postictal cerebral blood flow as assessed by perfusion‐weighted MRI. Bayesian generalized mixed‐effects models were applied for analyses.

**Results:**

We included 300 seizures, postictal EEGs, and reorientation time values, and 76 MRI perfusion measures from 33 patients (median age 53 years, 19 female). Pretreatment with acetaminophen or nimodipine was not associated with change in speed of EEG recovery compared to placebo (1.13 [95%CI 0.92, 1.40] and 1.07 [95%CI 0.87, 1.31], respectively), nor with the secondary outcomes. No patient reached full EEG recovery at 1 h post‐seizure, despite clinical recovery in 89%. Longer seizures were associated with slower EEG recovery and lower postictal perfusion. Nimodipine altered regional perfusion in the posterior cortex.

**Interpretation:**

Pretreatment with acetaminophen or nimodipine did not alleviate symptoms and signs of the postictal state. Systematic study of the postictal state after ECT‐induced seizures is feasible.

## Introduction

The postictal state has been recognized as a neglected entity in the management of epilepsy.^1^, ^2^ After seizures, neurological phenomena, cognitive deficits, and psychiatric symptoms may occur, that strongly add to the burden and morbidity of epilepsy. The exact pathophysiology of the postictal state remains unclear, but postictal cerebral hypoperfusion through (local) vasoconstriction is associated.^3^, ^4^, ^5^ In an animal model, postictal behavioral symptoms were strongly related to local cerebral arteriolar vasoconstriction, resulting in a drop of local brain tissue oxygenation and cerebral perfusion. This could be prevented by targeting cyclooxygenase (COX)‐2 or L‐type calcium‐channels.^4^, ^5^ Rats pretreated with acetaminophen (i.e., a selective COX‐2 inhibitor) or nifedipine (i.e., a calcium‐channel blocker) showed diminished postictal behavioral symptoms.^4^, ^5^ Because acetaminophen and nifedipine are safe and widely available, these regular medications offer an opportunity for prophylaxis or treatment of postictal symptoms in humans.

Assessing the postictal state is not straightforward, because of the unpredictable nature of seizures in epilepsy patients. Electroconvulsive therapy (ECT) provides a unique possibility to study the postictal state in a well‐controlled, reproducible environment. ECT is regularly used in patients with severe depression.^6^, ^7^, ^8^, ^9^ During this treatment, electrically induced generalized tonic–clonic seizures are followed by acute postictal side effects, similar to those in epilepsy patients.^6^, ^10^ As a clinical measure of the postictal state, the time it takes for patients to reorient in person, place, and time can be assessed.^11^ Furthermore, continuous electroencephalography (EEG) before, during, and after ECT‐induced seizures can provide quantitative features for postictal recovery.^6^, ^12^ Arterial spin labeling magnetic resonance imaging (ASL‐MRI) enables the measurement of postictal cerebral blood flow (CBF).^13^ These assessments may provide new insights into clinical, electrophysiological, and brain perfusion characteristics of the postictal state.

We aimed to investigate the effects of acetaminophen or nimodipine on the postictal state in patients with ECT‐induced seizures. Nimodipine was chosen instead of nifedipine because it acts more specifically in dilating cerebral blood vessels.^14^ Furthermore, we sought to elucidate electrophysiological and cerebral perfusion characteristics of the postictal state. To this end, we performed a prospective randomized clinical trial with three‐condition crossover design, with postictal EEG and MRI as measures of outcome.

## Methods

### Trial design

The StudY of effect of Nimodipine and Acetaminophen on Postictal Symptoms after Electroconvulsive therapy (SYNAPSE) trial (https://clinicaltrials.gov/study/NCT04028596) was a prospective, monocenter, single‐blind, clinical trial with a three‐condition randomized crossover design, executed at Rijnstate Hospital, Arnhem, The Netherlands. The trial was approved by the local medical‐ethical authority and conducted according to standards of good clinical practice. Exact trial details are described elsewhere.^15^

### Trial population

Patients indicated for ECT because of severe depressive episodes, and who were willing and able to provide written informed consent, were eligible for inclusion. Patients with known adverse or allergic reactions to acetaminophen or nimodipine, chronic use of acetaminophen, calcium antagonists, or nonsteroidal anti‐inflammatory drugs, and contraindications for undergoing MRI were excluded.

### Randomization and masking

Before inclusion, four blocks of each three interventions were randomly computer‐generated by an external hospital party and allocated sequentially. The allocation sequence was concealed in a closed closet, not accessible by the investigator performing inclusion.^15^ Patients were enrolled by the treating psychiatrist. Interventions were assigned by a coordinating physician who was not involved in data acquisition. Outcome assessors were blinded to the treatment allocation. Patients were partly blinded, as they could recognize the differences between the treatment conditions.

### Procedures

ECT was performed in accordance with internationally accepted clinical guidelines, using unilateral (UL) and bilateral (BL) electrode placements with titration‐ or age‐based dosage methods, and under proper anesthesia and muscle relaxation.^15^, ^16^ At a maximum of 2 h prior to each ECT session, patients received one of three treatment conditions in random and counterbalanced order (i.e., 50 cc of only water, 50 cc of water with 60 mg nimodipine, or 50 cc of water with 1000 mg acetaminophen). The first ECT session served as the baseline measurement and was used to collect clinical and EEG data without exposure to any of the treatment conditions.

### Primary outcome measure

Continuous EEG was monitored before, during, and 1 h following each ECT session. The primary outcome measure was “*speed* of postictal EEG recovery” (quantified with a time constant τ; see Supplementary Methods, Fig. S2, and our earlier work).^12^ This measure was obtained by fitting an exponential function to the postictal EEG data using a modified version of the temporal brain symmetry index (tBSI).^17^ Larger values of τ indicate slower postictal recovery.

### Secondary outcome measures

Secondary outcomes were the “*extent* of postictal EEG recovery” at 1 h, clinical reorientation time (ROT), and postictal CBF.

The extent of postictal EEG recovery at 1 h compared to baseline was quantified with the temporal BSI, as described previously (Supplementary Methods).^12^ ΔBSI values range from 0 to 1, where larger values indicate a greater difference between the baseline and the postictal state, reflecting more enduring postictal EEG disturbances.

To assess the postictal state clinically, we used the ROT questionnaire.^11^, ^18^ In 5‐minute intervals, patients were asked questions about their orientation in person, place, and time. The ROT was defined as the time (in minutes) between seizure onset and the first instance in which the patient correctly answered at least four out of five questions (relative to their baseline). If a patient was not reoriented within 100 min, the maximum ROT score was assigned (i.e., 100 min).

ASL‐MRI data were collected before the ECT course (i.e., baseline), and after three different ECT sessions, one in each experimental condition (see Supplementary Methods for details of preprocessing and elsewhere^19^). Postictal CBF was obtained by ASL‐MRI ~1 h after administration of the ECT stimulus. Postictal *global* and *regional* CBF were calculated to investigate the magnitude of CBF differences (ΔCBF) between the treatment conditions, respective to baseline. In advance, we selected 10 regions of interest (ROI) that had shown postictal CBF changes in earlier studies.^20^, ^21^, ^22^, ^23^

### Statistical analyses

#### Power calculation

The sample size was calculated in advance of the study, based on a mixed model with repeated measurements with an estimated effect size of 0.25, a type 1 error rate of 5%, and a correlation of 0.4 between measurements, resulting in a total of 33 patients with at least 12 repeated seizures to achieve a power of 0.80.^24^

All statistical analyses were conducted according to the predefined and published statistical analysis plan for SYNAPSE (NCT04028496; ClinicalTrials.gov).^24^ Numbers and percentages are presented for categorical variables and medians and interquartile ranges (IQR) for continuous variables. We checked for completeness of all relevant data ahead of unblinding. No imputation of missing data was performed. Weakly informative priors with a mean of 0 and standard deviation of 50 were included in all Bayesian models for all predictors, using R (version 4.2.3) with brms package.^25^, ^26^

#### Speed of postictal EEG recovery

We fitted a Bayesian generalized mixed‐effects model with random intercept and random slope. All values of our primary outcome variable, the time constant τ, were modeled with a lognormal distribution. Logarithm and logit links were used for linearization.^27^ Fixed effects were the interventions (i.e., acetaminophen, nimodipine, and placebo), age, sex, time (i.e., number of the ECT session), and electrode placement (i.e., UL or BL). Acetaminophen or nimodipine were compared to the placebo condition, which served as the reference. Random effects were patient ID, intervention, and time, to account for within‐patient variability that might be partly determined by time and intervention effects.

A post hoc model was developed to compare treatment (i.e., both active interventions pooled) versus no treatment (i.e., placebo) for speed of postictal EEG recovery. A region of practical equivalence (ROPE) analysis was conducted to test the hypothesis of no practically relevant effect of the study medications. This hypothesis was accepted if 95% of the posterior distribution of the main contrasts (study medication versus placebo) fell in the range of −0.1 to 0.1 standard deviation.^28^

#### Extent of postictal EEG recovery and clinical reorientation time

Bayesian generalized mixed‐effects models with random intercept and random slope were fitted for the secondary outcome variables ΔBSI and ROT, modeled with beta distributions. The same fixed effects were included, followed by the same post hoc models to compare treatment versus no treatment. Parameter estimates and credibility intervals are presented as multiplication factors, where numbers larger (or smaller) than 1 indicate a positive (or negative) effect. Multiplication factors are inherent to generalized models because of the utilized link function, which transforms the parameter estimates to the log scale. The estimates were exponentially transformed for interpretability.^27^ This allowed us to interpret the influence of the interventions with a clear reference (e.g., a credible multiplication factor 1.13 for acetaminophen versus placebo would mean that acetaminophen leads to a 13% longer postictal EEG recovery compared to placebo).

#### Postictal cerebral blood flow

Postictal change from baseline in mean *global* and *regional* CBF (ΔgCBF and ΔrCBF, respectively) were compared between all interventions in Bayesian random intercept mixed‐effects models, with Gaussian response distributions. Fixed effects were intervention, age, sex, electrode placement, and the time interval between ECT stimulus and the start of the ASL‐MRI sequence. Because of the previously established relationship between seizure duration and postictal CBF changes, seizure duration was added as fixed effect.^19^ Random effect was patient ID.

In addition, a full‐factorial voxel‐wise repeated measures analysis of variance was performed to explore the effects of the interventions on ΔCBF. Post hoc comparisons were performed after a significant omnibus *F*‐test (i.e., acetaminophen versus placebo, nimodipine versus placebo, acetaminophen versus nimodipine). Age, sex, electrode placement, the time interval between ECT stimulus and the start of the ASL‐MRI sequence, and seizure duration were entered as covariates of no interest. Voxel‐wise tests were considered significant, if these were family wise error cluster‐level corrected (*p* < 0.05, or for post hoc comparisons *p* < 0.017) at a height threshold of *p* < 0.001. Finally, post hoc mixed‐effect models were used to investigate the relationship between ROT and ΔgCBF and ΔrCBF, because we expected that more clinical disorientation would be associated with more severe global or regional postictal hypoperfusion.^5^

## Results

### Included sample and available measurements

**Figure 1:**
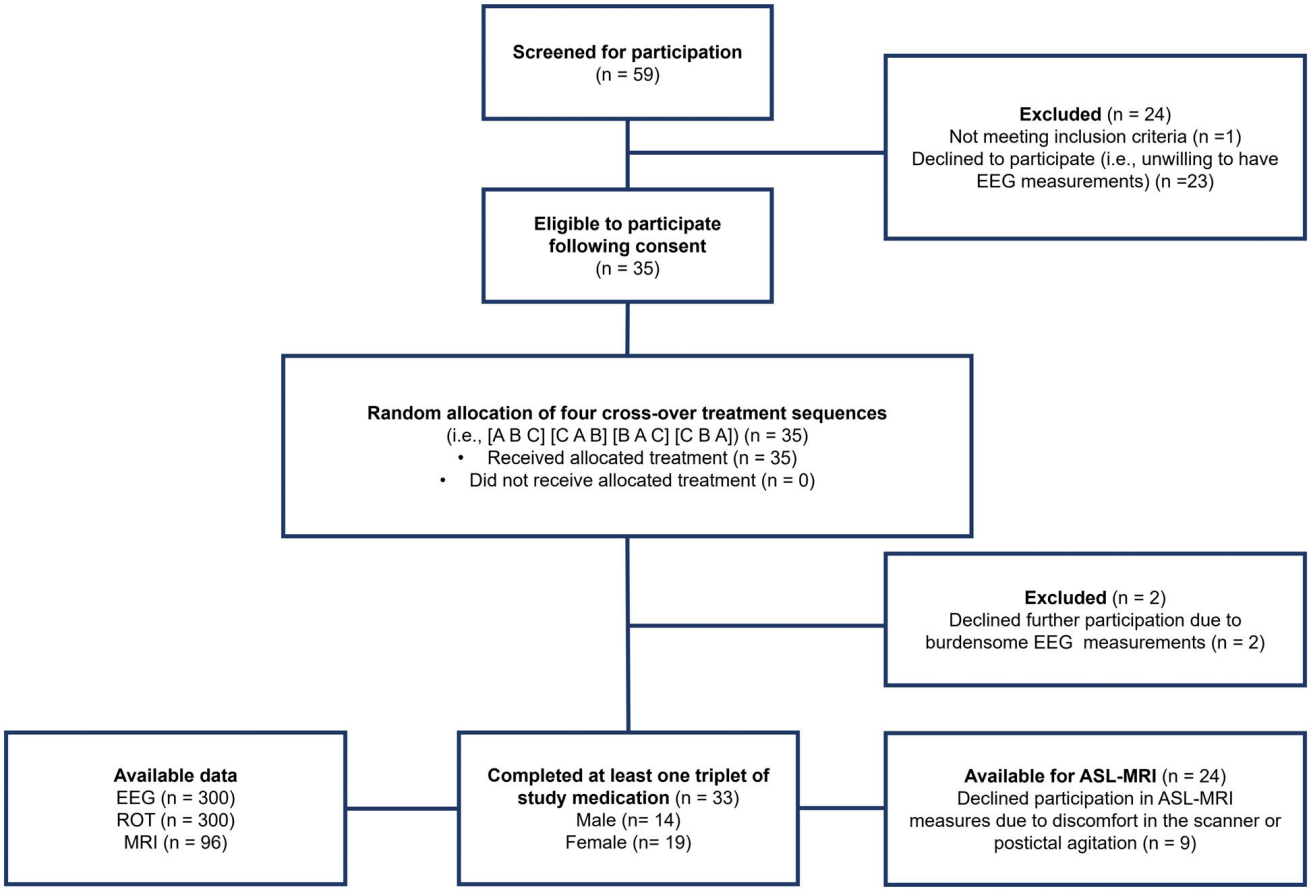
Trial profile. ASL‐MRI, arterial spin labeling MRI; EEG, electroencephalography; MRI, magnetic resonance imaging; ROT, reorientation time.

In total, we collected 328 EEGs, 328 ROT, and 96 ASL‐MRI measurements from 33 patients between December 3, 2019, and April 12, 2023. Out of these, 300 EEGs, 300 ROT values, and 96 ASL‐MRI scans from 24 patients were included in our final analyses (per patient, median number of postictal EEG and ROT measures = 9, IQR 3, range 3–14; Fig. 1).

Table 1 presents the patient, ECT, and trial characteristics of the included sample. Median age was 53 (IQR 21.3) years, and 19 patients were female (56%). Most patients (*n* = 24, 73%) were treated with BL ECT, and concomitant medication use was as expected.

**Table 1:** Patient, electroconvulsive therapy (ECT), and trial characteristics of the SYNAPSE trial.

	Total sample (*n* = 33)	Patient population for ASL‐MRI analyses (*n* = 24)
*Patient characteristics*		
Age in years, median (IQR; range)	53 (21.3; 24–82)	56 (22.5; 24–82)
Female sex, *n* (%)	19/33 (56)	15/24 (63)
*ECT characteristics*		
Bilateral electrode placement at the end of the ECT course, *n* (%)	24/33 (73)	15/24 (63)
Electrical charges to elicit seizures in milliCoulombs, median (IQR; range)	304.7 (228.8; 125.6–813.0)	304.4 (250.2; 125.6–813.0)
Seizure duration of all included ECT sessions during the ECT course in seconds, median (IQR; range)	51 (25.5; 16.3–178.2)	55 (24.5; 19.6–140.1)
Total number of ECT sessions during the ECT course, median (IQR; range)	12 (9; 7–100)	12 (8; 8–100)
Concomitant psychopharmacological drug use during the ECT course, *n* (%)		
Antidepressants	24/33 (73)	18/24 (75)
Antipsychotics	23/33 (70)	17/24 (71)
Antiepileptics	7/33 (21)	4/24 (17)
Benzodiazepines	20/33 (61)	17/24 (71)
Lithiumcarbonate	2/33 (1)	2/24 (8)
Number of patients needing medication for severe postictal symptoms after ECT*, *n* (%)	12/33 (36)	8/24 (33)
*Trial characteristics*		
Number of EEGs/ASL‐MRI scans per intervention, *n* (%)		
Acetaminophen	100/300 (33)	24/72 (33)
Nimodipine	99/300 (33)	24/72 (33)
Placebo	101/300 (34)	24/72 (33)
Interval between administration of study medication and application of ECT stimulus in minutes, median (IQR; range)**		
Acetaminophen	145.5 (31.8; 221–50)	144.5 (27; 212–93)
Nimodipine	137 (36; 265–88)	136 (24; 265–100)
Placebo	147 (44; 245–77)	146 (34.5; 245–77)
Interval between application of the ECT stimulus and postictal ASL‐MRI acquisition in minutes, median (IQR; range)	NA	64 (15; 35–94)

ASL, arterial spin labeling; BL, bifrontotemporal; EEG, electroencephalography; ECT, electroconvulsive therapy; IQR, inter quartile range; EEG, electroencephalogram; MRI, magnetic resonance imaging; NA, not applicable.

* Postictal medication consisted of a single dose of midazolam, ranging between 2 and 5 mg.

** Differences in medication administration were tested statistically between the interventions and revealed no significant differences (*p* = 0.482).

### Primary outcome measure – Speed of EEG recovery

**Figure 2:**
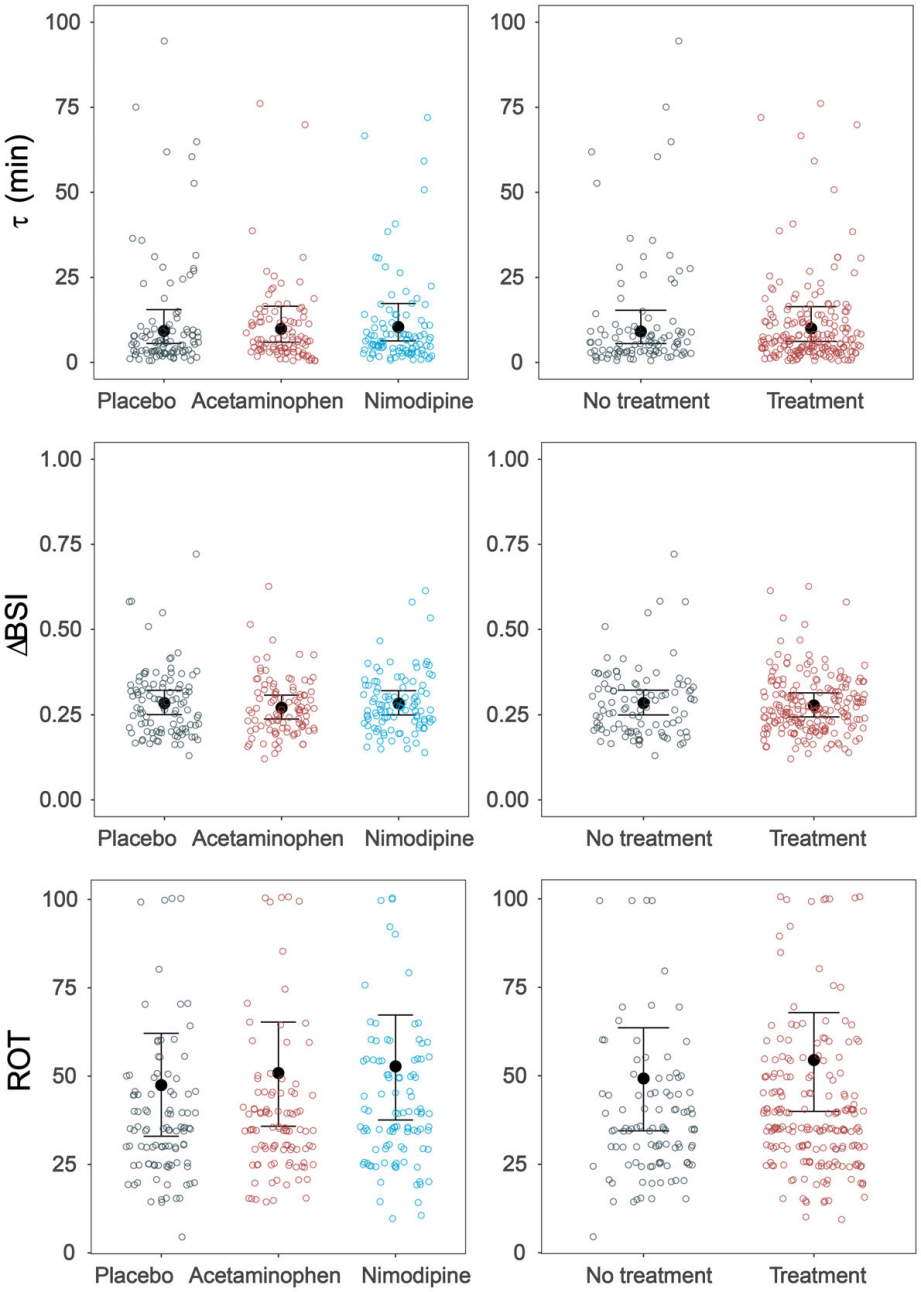
Overview of main findings. The left panels show the three conditions (i.e., placebo, acetaminophen, and nimodipine), and the right panels show no treatment (i.e., placebo) and treatment (i.e., acetaminophen or nimodipine). Upper panels: time constants τ, in minutes, *speed* of postictal EEG recovery. Middle panels: *extent* of postictal EEG recovery (ΔBSI). Lower panels: clinical reorientation time (ROT), in minutes. None of the Bayesian models revealed credible differences between any of the conditions (i.e., indicated by black credibility intervals). Details are presented in Table S2.

Overall, the median *speed* of postictal EEG recovery (quantified as the time constant τ) was 6.0 min (IQR 8.6 min) and ranged from 0.5 to 94 min. Medians of *speed* of postictal EEG recovery after treatment with acetaminophen, nimodipine, and placebo were 6.2 min (IQR 8.1), 6.3 min (IQR 9.3), and 5.8 min (IQR 7.0), respectively (Fig. 2). Bayesian analyses revealed no effects on *speed* postictal EEG recovery of acetaminophen or nimodipine (1.13 [95% CI 0.92, 1.40] or 1.07 [95% CI 0.87, 1.31], respectively; Table 2; Fig. 2, upper left panel), nor of any active treatment (1.10 [95% CI 0.91, 1.31], Fig. 2, upper right panel).

**Table 2:** Results of Bayesian mixed‐effects models.

							Equivalence test Acetaminophen versus Placebo and Nimodipine versus Placebo	Equivalence test Treatment versus placebo
*Predictors*	**Speed of postictal EEG recovery (τ, min)**						*ROPE [−1.4, 1.4]*	
	*Estimate*	*CI95 lower*	*CI95 upper*	*Estimate*	*CI95 lower*	*CI95 upper*		
Intercept	10.42	2.65	40.14	10.56	2.80	39.69		
Acetaminophen versus Placebo	1.13	0.92	1.40				Accepted	
Nimodipine versus Placebo	1.07	0.87	1.31				Accepted	
Treatment versus Placebo				1.10	0.91	1.31		Accepted
	**Extent of postictal EEG recovery (ΔBSI)**						*ROPE [−0.01, 0.01]*	
	*Estimate*	*CI95 lower*	*CI95 upper*	*Estimate*	*CI95 lower*	*CI95 upper*		
Intercept	0.42	0.26	0.65	0.42	0.27	0.66		
Acetaminophen versus Placebo	0.99	0.89	1.09				Undecided	
Nimodipine versus Placebo	0.93	0.84	1.03				Undecided	
Treatment versus Placebo				0.96	0.88	1.05		Undecided
	**Clinical reorientation time (ROT, min)**						*ROPE [−1.9, 1.9]*	
	*Estimate*	*CI95 lower*	*CI95 upper*	*Estimate*	*CI95 lower*	*CI95 upper*		
Intercept	0.72	0.14	3.75	0.83	0.16	4.39		
Acetaminophen versus Placebo	1.24	0.86	1.77				Accepted	
Nimodipine versus Placebo	1.15	0.90	1.47				Accepted	
Treatment versus Placebo				1.24	0.99	1.55		Accepted
	**Change in postictal *global* CBF (gCBF, mL/100 g/min)**						*ROPE [−1.7, 1.7]*	
	*Estimate*	*CI95 lower*	*CI95 upper*					
Intercept	−8.38	−31.27	14.46					
Acetaminophen versus Placebo	2.47	−1.36	6.24				Accepted	
Nimodipine versus Placebo	−2.12	−5.69	1.46				Accepted	

Bayesian analyses were performed twice, with the same outcome variables, however, with an additional fixed effect active treatment (i.e., acetaminophen or nimodipine) versus placebo. However, it was not possible to run the analysis on treatment vs placebo for postictal global CBF as both acetaminophen or nimodipine had opposite effects. Fixed effect time refers to the number of the ECT session. Empty cells indicate that fixed effects were not included in the respective model; Accepted = The posterior distribution falls completely within the ROPE, leading to acceptance of the null hypothesis (i.e., there is no effect on the outcome measure); Undecided = The posterior distribution falls partly within the ROPE, which prevents definitive conclusion.

CBF, cerebral blood flow; CI, credibility interval; EEG, electroencephalography; ROPE, regional of practical equivalence.

Time constant τ was associated with ROT (1.004 min [CI95 1.000, 1.010]), the number of previous ECT sessions (1.040 [CI95 1.005, 1.077]), and seizure duration (1.009 [1.004, 1.014]), indicating slower EEG recovery in patients with longer ROT, more previous ECT sessions, or longer seizures. Other variables age (0.995 [CI95 0.975, 1.016]), sex (0.919 [CI95 0.490, 1.717]), electrode placement (0.809 [CI95 0.498, 1.300]), or use of postictal benzodiazepines (1.211 [CI95 0.614, 2.349]) were unrelated with the time constant τ.

### Secondary outcome measures

#### Extent of EEG recovery

None of the patients recovered postictally toward EEG baseline levels within 1 h in any of the 300 EEGs (i.e., ΔBSI = 1), indicating that no full recovery was reached at 1 h after the seizure. Median ΔBSI appeared 0.73 (IQR 0.1) in the total sample. After pretreatment with acetaminophen, nimodipine, and placebo, medians of ΔBSI were 0.73 (IQR 0.1), 0.74 (IQR 0.1), and 0.73 (IQR 0.1), respectively. Bayesian analyses revealed no effects on ΔBSI of acetaminophen or nimodipine, nor of any active treatment (Table 2, Table S2, and Fig. 2, middle panel).

ΔBSI was associated with seizure duration (0.997 [CI95 0.995, 0.999]), indicating less EEG recovery in patients with longer seizures. ΔBSI was not associated with age (1.000 [CI95 0.992, 1.007]), sex (1.093 [CI95 0.860, 1.382]), electrode placement (1.019 [CI95 0.830, 1.233]), use of postictal benzodiazepines (1.167 [CI95 0.912, 1.495]), ROT (1.000 [CI95 0.992, 1.007]), or number of previous ECT sessions (1.013 [CI95 0.994, 1.032]).

#### Reorientation time

Median ROT was 35 min (IQR = 21.5 min) and ranged from 5 to 100 min. After pretreatment with acetaminophen, nimodipine, and placebo, the medians of ROT were 40 min (IQR 25), 35 min (IQR 15), and 35 min (IQR 20), respectively. Bayesian analyses revealed no effects of acetaminophen or nimodipine, and neither of any treatment versus placebo (Table 1, Table S2, and Fig. 2, lower panel). In 268 of 300 postictal states (89%), complete clinical ROT recovery appeared within 1 h, while EEGs of those patients were still perturbed (i.e., no full EEG recovery to baseline).

#### Cerebral blood flow

**Figure 3:**
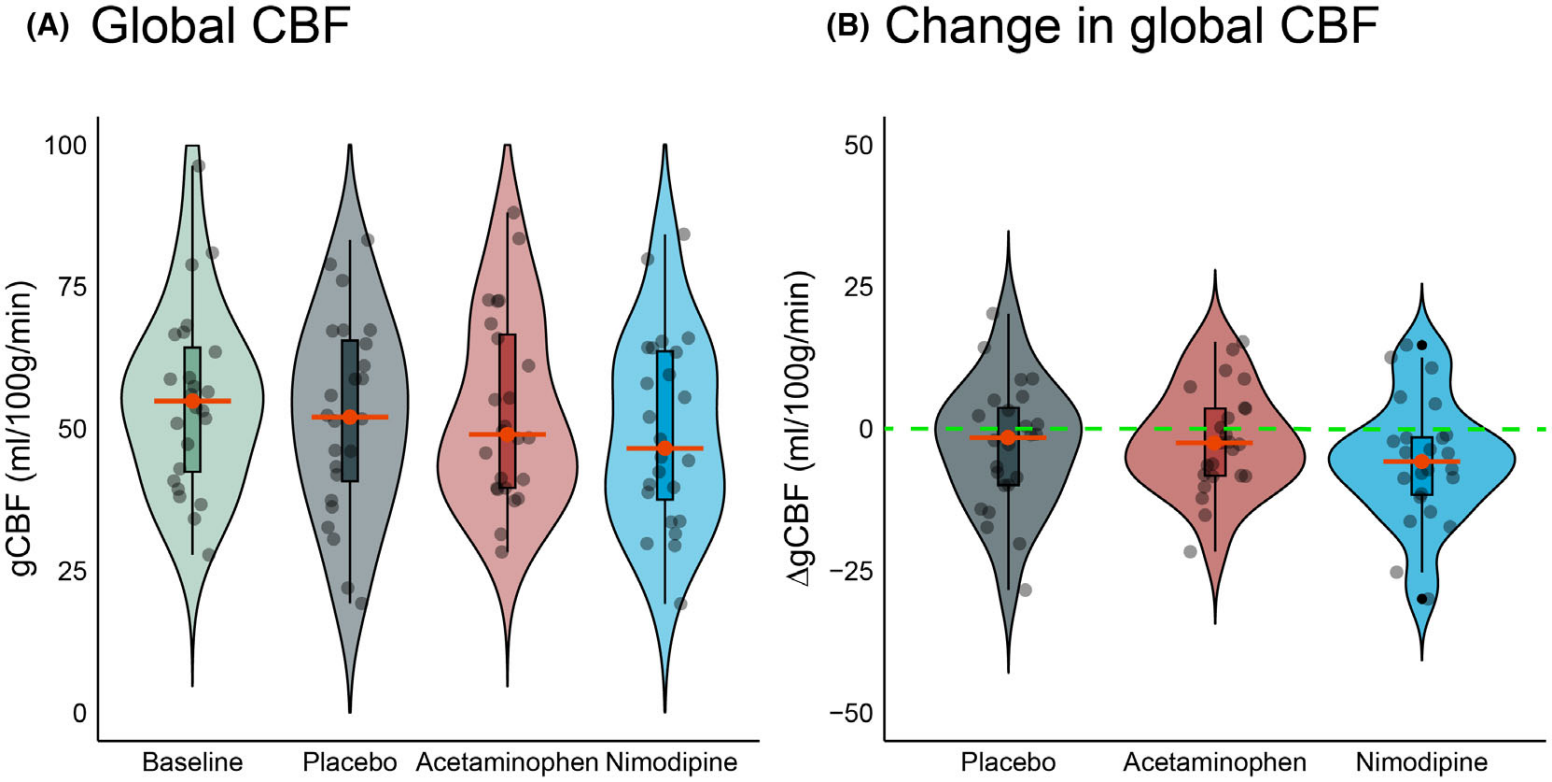
Postictal *global* cerebral blood flow (gCBF) median values (in mL/100 g/min) were comparable for acetaminophen, nimodipine, and placebo treatment. (A) gCBF values at baseline and in the postictal state with placebo, acetaminophen, or nimodipine treatment. (B) Change in gCBF (ΔgCBF) after placebo, acetaminophen, or nimodipine treatment. The dashed green line indicates no change of postictal CBF with respect to baseline. Red lines indicate median (change in) CBF.

The median time interval between ECT stimulus and the start of the ASL‐MRI sequence was 64 minutes (IQR 15, range = 35–94). In the total sample, median baseline gCBF was 55.2 mL/100 g/min (IQR 22.5), median postictal gCBF was 49.7 mL/100 g/min (IQR 25.1), and median ΔgCBF was −3.0 mL/100 g/min (IQR 12.5). After pretreatment with acetaminophen, median postictal gCBF was 49 mL/100 g/min (IQR 27), with nimodipine 46.6 mL/100 g/min (IQR 26.1), and in the placebo condition 52 mL/100 g/min (IQR 24.8) (Fig. 3A). Median postictal ΔgCBF did not differ between the treatment conditions (Table 1, Table S2 and S3).

Perfusion outcomes varied between patients. For example, we observed clear postictal hypoperfusion in one patient after pretreatment with nimodipine, with *global* CBF decreases up to 47% respective to baseline (i.e., up to 30 mL/100 g/min); another patient had postictal *global* CBF increases up to 48% compared to baseline after both interventions and placebo. Age was negatively associated with baseline gCBF (−0.6 mL/100 g/min [CI95 –1, −0.2]). As we previously found a relationship between seizure duration and ΔgCBF, we investigated this relationship and compared it in all intervention groups.^19^ The median seizure duration with acetaminophen, nimodipine, or placebo pretreatment was 55 sec (IQR = 26, range = 20–112), 54 sec (IQR = 22, range = 25–140), and 55 sec (IQR = 18, range = 25–114), respectively (Fig. S5A). There was no association between ΔgCBF and seizure duration or an interaction between seizure duration and the effect of the intervention (Fig. S5B, Table S6). None of the other covariates had a relationship with ΔgCBF (i.e., time to ASL‐MRI acquisition, age, sex, electrode placement, number of previous ECT sessions, and ROT).

Spatial analysis (omnibus voxel‐wise *F*‐test) of postictal CBF changes revealed significant effects in the left precuneus (*p* = 0.030) and the right superior parietal lobule (*p* = 0.014), indicating significant postictal hypoperfusion in these regions. Post hoc comparisons revealed that pretreatment with nimodipine showed more postictal CBF decreases in one cluster in the precuneus, compared to placebo. Pretreatment with acetaminophen showed increased postictal CBF, compared to nimodipine, in one cluster in the precuneus, and in two clusters in the superior parietal lobule (Fig. S6 and Table S4). In exploratory analyses, we did not find any relationship between ROT and ΔrCBF (Table S5).

## Discussion

This is the first randomized clinical trial aimed at alleviating symptoms in the postictal state after seizures. In patients with ECT‐induced seizures, pretreatment with acetaminophen or nimodipine did not improve the *speed* or *extent* of postictal EEG recovery, nor the clinical reorientation time or postictal global CBF. EEG appeared more sensitive than ROT to measure postictal recovery: While most postictal patients reached complete clinical reorientation, none reached EEG recovery within 1 h after the seizure. Less postictal EEG recovery occurred in patients with more seizures and longer seizure duration. Patients showed large variations in postictal cerebral perfusion between measurements. Pretreatment with acetaminophen or nimodipine induced very local alternations of postictal cerebral perfusion in the precuneus and superior parietal lobule. Additionally, this study shows that studying interventions to influence the postictal state is feasible in ECT patients.

To date, there is no gold standard to study the postictal state. We applied a combination of clinical, EEG, and MRI measures to examine the effects of potential prophylactic treatments for improving postictal symptoms after ECT‐induced seizures. Postictal hypoperfusion and hypoxia have been previously shown to be involved as the underlying pathophysiological mechanism of the postictal state in animal models.^5^ We expected that our EEG measure, the temporal brain symmetry index, would be sensitive to hypoxic brain changes because the observed postictal EEG changes are similar to those observed in patients with cerebral ischemia.^29^, ^30^, ^31^, ^32^ We indeed observed EEG changes and variations in postictal CBF, but our experimental interventions did not lead to improved EEG recovery, shorter clinical reorientation time, or better postictal perfusion.

Still, we enhanced our understanding of electrophysiology and cerebral perfusion in the postictal state. First, we show that, although patients may look conscious and oriented 1 h after the seizure, their electrophysiological state and cerebral perfusion may still be affected. Second, EEG abnormalities persist beyond 1 h after the seizure and relate to a presumed cumulative effect of multiple previous seizures. These EEG findings suggest that restoration of orientation probably depends on gradual cortical synaptic recovery, with more seizures leading to longer postsynaptic suppression, in line with previous findings.^33^

Postictal cerebral hypoperfusion was observed in some patients in all conditions, while other patients had postictal hyperperfusion. Pretreatment with nimodipine seemed to be related to decreased rather than increased *global* postictal perfusion, which contradicted our hypothesis. Furthermore, the spatial distribution of changes in perfusion varied: Our voxel‐wise analyses showed postictal CBF decrease after pretreatment with nimodipine in the precuneus, and – otherwise – after pretreatment with acetaminophen an increase in the superior parietal lobule. These divergent individual findings are intriguing and may result from patient‐specific factors (such as age or brain tissue differences), ECT‐related variables (e.g., electrode placement, anesthesia, and concomitant medication use), methodological limitations (e.g., technical ASL‐MRI failures or differences in timing), or simply random fluctuations. Although we corrected for age in all our statistical models, age appeared negatively associated with global CBF at baseline, which may have influenced the individual vulnerability for (local) hypoperfusion. Also, individual brain anatomy may determine the level of tissue penetration of the ECT stimulus, which may have resulted in variable current exposure and consecutive (local) brain perfusion.^34^ However, given the (sometimes) impressive regional postictal cerebral perfusion alterations, it is still remarkable that no associations were shown with our clinical and EEG measures.

Several explanations are possible for the lack of effects in our sample. First, the human pathophysiology of the postictal state may differ from those in rats, which may have hampered the translation to the human model. Additionally, severely depressed patients show different (baseline) EEGs and cerebral perfusion measures compared to healthy individuals^35^, ^36^ and all ECT patients were anesthetized before seizure‐induction.^37^ This may affect our clinical‐, EEG‐, and ASL‐MRI measures. Furthermore, electrode placements, administered electrical charges, and elicited seizure durations differed per patient, which may have resulted in different seizure onset zones, seizure propagations, and postictal cerebral perfusion patterns.^19^ While it is true that postictal blood flow changes were variable in these patients, changes in epilepsy patients are equally variable, with only local changes in differing seizure onset zones.^5^ It may be possible that ECT‐induced seizures slightly vary in their seizure onset zones, depending on the distribution of the electric field, which depends on electrode placement and individual differences in skull thickness.^34^ Unfortunately, the size of the subsets of our patients to analyze discrete regional effects of these different characteristics was too small. In comparison with the original animal model, our applied dosages of acetaminophen and nimodipine were likely to be modest and the method of administration differed substantially.^5^ The oral administration may have led to insufficient blood concentrations in the central nervous system and may have caused considerable variability in intracerebral concentrations between patients. Unfortunately, we did not collect blood samples, but nimodipine concentrations after 2 h of administration may range between 10 and 35 ng/mL.^38^ The effect of a single dose in rats was so large that it was reasonable to test a single dose in humans compared to pretreating patients for several days. In the animal model, 20 mg/kg dosed ibuprofen showed similar effects as acetaminophen on inhibiting severe hypoxia.^5^ Because these dosage levels are normal for human use (compared to the excessive human dosage of acetaminophen used in the animals), ibuprofen may serve as a new candidate drug. Future studies may address this.

The strengths of our study include its prospective randomized crossover design and the systematic collection of 300 EEGs, 300 ROTs, and 96 ASL‐MRI scans. We show that it is feasible to study the postictal state in patients with ECT‐induced seizures. Although our study was sufficiently powered to show the influence of the interventions on the speed of EEG recovery, the moderate sample size (*n* = 33 patients) impacted our ability to detect meaningful differences in our secondary analyses. Due to the naturalistic sample of ECT patients (e.g., with different concomitant medication use, medical comorbidities, postictal medication use), we had hoped to overcome the confounding factors by using each patient as their own control, but this may not have been sufficient to detect possible differences in postictal recovery. Furthermore, the postictal ASL‐MRI scans may have been performed too late to capture the expected acute perfusion effects.^20^, ^37^ Moreover, correcting for test–retest variability, as we showed earlier in ASL‐MRI analyses of our placebo condition,^19^ may limit the sensitivity to detect postictal perfusion alterations.

In conclusion, we showed that pretreatment of ECT patients with standard dosages of acetaminophen or nimodipine did not improve the *speed* or *extent* of postictal EEG recovery, the clinical reorientation time, or postictal global cerebral perfusion. Nevertheless, our prospective study shows that it is feasible to systematically study the postictal state after ECT‐induced seizures, including interventions to alleviate postictal symptoms and signs. EEG is a sensitive outcome measure for postictal recovery. Our findings open avenues for new observational and intervention studies of the postictal state.

## Funding Information

This work has been funded by EpilepsieNL (grant number WAR 19‐02).

## Author Contributions

J. C. M. Pottkämper, PhD involved in the investigation, data curation, software, methodology, project administration, formal analyses, visualization, and writing (original draft). J. P. J. A. Verdijk, MD involved in conceptualization, project administration, methodology, and writing (original draft). S. Stuiver, MSc involved in investigation, software, data curation, and writing (original draft). E. Aalbregt, MSc involved in investigation, software, and writing (original draft). F. ten Doesschate, PhD involved in supervision, software, validation, and writing (original draft). Dr. E. Verwijk, PhD involved in conceptualization and writing (original draft). Dr. M. Schmettow, PhD involved in methodology, software, formal analyses, supervision, and writing (original draft). G. A. van Wingen, PhD involved in conceptualization, supervision, and writing (original draft). M. J. A. M. van Putten, MD PhD involved in conceptualization, funding acquisition, resources, and writing (original draft). J. Hofmeijer, MD PhD involved in conceptualization, funding acquisition, resources, and writing (original draft). J. A. van Waarde, MD PhD involved in conceptualization, funding acquisition, resources, and writing (original draft).

## Conflict of Interest

The authors declare no competing interests.

## Supporting information


Data S1.

## Data Availability

Individual participant data that underlie the results reported in this article, after de‐identification (text, tables, figures, and appendices) will be available including data dictionaries, as well as the study protocol and statistical analysis plan. Data will be available following article publication to researchers who provide a methodologically sound proposal to achieve the objectives in the approved proposal. MRI and clinical data will be uploaded to the database of the Global ECT‐MRI Research Collaboration (GEMRIC; www.gemric.org), which consortium has procedures for other researchers to use these data. EEG data will be uploaded to a server at the University of Twente. Proposals should be directed to the principal investigator of SYNAPSE (jvanwaarde@rijnstate.nl). To gain access, data requestors will need to sign a data access agreement.
