# Supplementary material for: Exploring postictal recovery with acetaminophen or nimodipine: A randomized‐controlled crossover trial
Source: Ann Clin Transl Neurol. 2024 Aug 19;11(9):2289–300. doi: 10.1002/acn3.52143 (PMC11537141; doi:10.1002/acn3.52143)
Supplement: Supplementary file 1 — Data S1. [file ACN3-11-2289-s001.docx]

**Supplementary material**

**eMethods**

**Electroconvulsive therapy (ECT)**

The positioning of ECT electrodes included right (RUL) and left (LUL) unilateral, or bifrontotemporal (BL, also named ‘bitemporal’) placement. A Thymatron System IV device (Somatics Incorporation Lake Bluff, Illinois, USA) was used to deliver ECT stimuli with a constant-current (0.9 Ampѐre), bidirectional, square wave, and brief pulse (1 ms). Patients were pre-oxygenated at 100% O_2_ prior to administration of the anesthetics (mostly etomidate, 0.2-0.3 mg/kg) and muscle relaxants (succinylcholine, 0.5-1 mg/kg). Positive pressure ventilation was maintained until the patient resumed spontaneous respiration following the ECT procedure. If needed, additional pre- (e.g., methyl-atropine, flumazenil) and post-ECT medication (i.e., midazolam 2.5-5 mg) were provided intravenously at the discretion of the treating psychiatrist and anesthesiologist, and were registered accordingly. Only one patient was so severely postictally agitated that a propofol bolus (i.e., 50 mg) was required. During the ECT-course, concomitant medication use remained constant and registered in the database (e.g., antidepressants, antipsychotics, benzodiazepines, somatic medication).

***Electroencephalogram (EEG) data acquisition, preprocessing, and outcome measure calculation***

*EEG acquisition.* We applied twenty silver/silver chloride cup electrodes with EC2 paste according to the international 10-20 system. EEGs were sampled at 256 Hz using a NeuroCenter EEG recording system (Clinical Science Systems) with a full-band DC amplifier (TMSi, Oldenzaal, The Netherlands). For patients treated with BL ECT electrode placement, F7, F8, T3, and T4 were positioned 10% above (F) or behind (T) the pre-defined position to provide room for ECT electrodes. ECT electrodes (i.e., right placement: F8 and T4) and Cz were adjusted in case of UL treatment. For pragmatic reasons, some EEG recordings were performed with a reduced montage with eleven electrodes, consisting of Fp1, Fp2, C3, C4, T3, T4, O1, O2, Fz, Cz, Pz, and the ground electrode. Impedances were kept below 5 kΩ. We recorded baseline resting-state EEGs of 5 min eyes closed prior to each ECT-session, followed by continuous EEG registration during the seizure up until 1 h of the postictal state.^18^

*EEG preprocessing*. Noisy EEG channels were excluded after manual inspection for artifacts. EEGs were band-ass filtered (1-25 Hz) using a first order Butterworth filter and converted to a bipolar montage. MATLAB R2021b was used for all preprocessing and analyses (MathWorks, Natick, MA, USA). Only in one patient, respiratory and muscle artifact contamination required a narrower band-pass filter (3-13 Hz) for further analyses.

*EEG primary outcome measure calculation, time constant τ***.** We used the temporal Brain Symmetry Index (tBSI) to characterize postictal EEG recovery.^19,20^ The tBSI is a measure to assess whole-brain temporal evolution with respect to a pre-defined baseline. In our study, the tBSI served as a metric of postictal EEG dynamics. As a baseline, we used the EEG that was recorded before each ECT-session. Welch’s averaged periodogram method was used to estimate spectral density. Normalized absolute spectral differences between artifact-free 5 second postictal epochs and baseline epoch (5 min eyes-closed) were calculated, using

$$tBSI=1- \frac{1}{N}\sum_{i=1}^{N} \frac{1}{K}\sum_{j=1}^{K} \left\| \frac{S_{i,j}-S_{ref, i, j}}{S_{i,j}+S_{ref, i, j}} \right\|$$

 (1)

where $S_{i,j}$ were Fourier coefficients belonging to frequency $i$ = 1, …, *N* of bipolar derivations $j$ = 1,2, …, *K* ^19^. $S_{i,j}$ were postictal segments and $S_{ref, i,j}$ were baseline segments. Postictal segments started from seizure offset and ended at the end of the recording (range = 30 – 60 min). Values range between [0,1], with tBSI=0 indicating maximal suppression and tBSI=1 return to baseline EEG (Figure S1).

To obtain a recovery curve that reflect the return of EEG activity, we subsequently fitted an exponential function to the mean of temporal evolution of the tBSI, given by

$a_{0}-a_{1} e^{-\frac{t}{\tau}}$ (2)

with parameters a_0_ (asymptote), a_1_, and a time constant *τ*. The time constant serves as a metric for the speed of EEG recovery. A cut-off of goodness-of-fit of 70% or higher was used to evaluate recovery curves as reliable. Larger values of τ reflect a slower postictal EEG recovery to its stationary value.^18^

*EEG secondary outcome measure calculation, ΔBSI*. Another measure to estimate postictal EEG recovery, i.e., the *extent* of recovery (how much postictal EEG characteristics deviate from baseline, see Figure S1), has been presented in previous work.^18^ The *extent* of postictal EEG recovery is reflected by extrapolating the BSI value to 60 minutes using the postictal curves, which is essentially the difference from baseline, ΔBSI. The ΔBSI has values between 0 and 1, where larger ΔBSI values indicate a greater difference between baseline and one hour postictally, reflecting enduring postictal EEG disturbances (i.e., less postictal recovery).

***Magnetic resonance imaging (MRI) data acquisition, preprocessing, and outcome measure calculation***

*MRI acquisition.* The T1-weighted (T1W) and resting-state ASL-MRI images were acquired using a 3T Philips Ingenia MRI scanner (Philips Healthcare, Best, The Netherlands) using a 15-channel head coil. T1W images were acquired with an isotropic voxel size of 1.1 mm; TR = 7.5 ms; TE = 4.6 ms; FOV = 256 x 238 mm², and 145 sagittal slices. Arterial spin labelling (ASL)-MRI images were acquired using pseudo-continuous ASL (pCASL) labeling and a 3D gradient-and-spin-echo readout module. The following scan parameters were used: labeling duration = 1800 ms, post label delay = 1900 ms, 4 background suppression pulses, repetition time (TR) = 4057 ms; echo time (TE) = 12 ms; flip angle = 90°; field of view (FOV) = 240 x 240 x 126 mm²; matrix size = 64 x 60; voxel size = 3 x 3 x 6 mm³; 21 transverse slices, no slice gap, scan duration = 5.5 min. The labeling planes were placed perpendicular to the distal ascending portions of the internal carotid.^42^ A total of four label-control pairs together with a M0 image (i.e., without labeling or background suppression) were acquired.

*MRI preprocessing*. FSL 6.0.3 (FMRIB Software Library, Functional Magnetic Resonance Imaging of the Brian Center, Department of Clinical Neurology, University of Oxford, Oxford, UK) and Statistical Parametric Mapping (SPM12; http://www.fil.ion.ucl.ac.uk/spm) were used in a Matlab R2022b environment (The MathWorks, Natick, MA, USA).^43^ T1W images were preprocessed using a standardized pipeline (i.e., fsl_anat) that included brain extraction, cortical and subcortical segmentation, and registration to standard space (i.e., Montreal Neurological Institute [MNI]).^14^ Individual gray matter (GM) probability maps were derived by considering voxels with a probability threshold > 0.2 to incorporate most gray matter voxels). These individual maps were binarized and combined to create a group-based binarized GM mask.

*Perfusion outcome measure calculation.* For ASL-MRI images, a mean perfusion image was created by subtracting the label images from the control images. Cerebral blood flow (CBF) was quantified using multicomponent modeling with Bayesian Inference for ASL-MRI (BASIL; http://fsl.fmrib.ox.ac.uk/fsl/fslwiki/BASIL).^44^ This involved kinetic model inversion, calibration with M0 scans, and registration using the oxford_asl function.^45^ Background suppression pulses have been applied to enhance the signal intensity of brain tissue.^14,46^ ASL-MRI images were registered to standard space via the T1W image, which resulted in a CBF gray matter image with a 2 mm isotropic resolution. For each patient, we derived a gray matter (GM) partial volume corrected and smoothed CBF map that were averaged to achieve a CBF map (eFigure 3). The GM partial volume images were multiplied with the group-based GM mask resulting in GM CBF images predominantly excluded white matter. Global CBF (gCBF) within the GM was calculated by averaging CBF values within each image. To assess changes in postictal gCBF compared to baseline (ΔgCBF) for each of the three experimental conditions (i.e., acetaminophen, nimodipine, placebo), difference images of baseline and postictal CBF maps were created. Mean perfusion of these difference images was calculated and used for mixed-effects models. Identical procedures were followed for creating regional CBF (rCBF) and changes in postictal rCBF compared to baseline (ΔrCBF) of the pre-slected regions-of-interest. Binary masks were created for all selected ROI’s,^24,25,47-50^ based on anatomical locations of the Talairach Daemon Labels, multiplied with individual partial volume corrected GM CBF images, and mean perfusion was calculated for each ROI.^51^


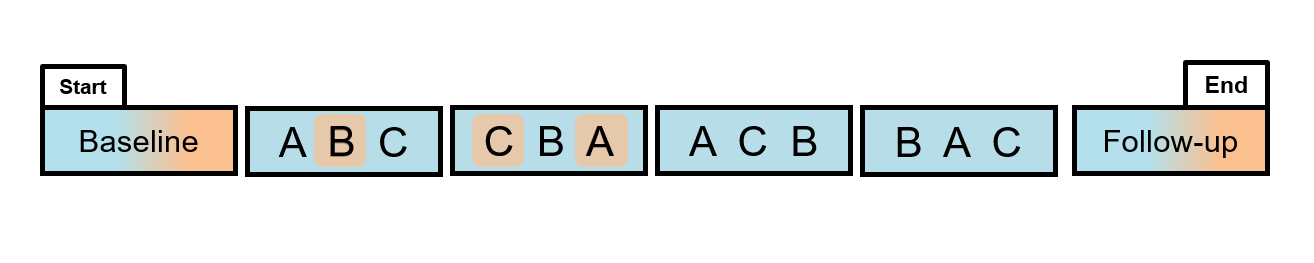


**Figure S1.** **Schematic representation of random and counterbalanced treatment allocation at each session of electroconvulsive therapy (ECT).** A, B, and C represent administration of 1000 mg acetaminophen, 60 mg nimodipine, and placebo condition (i.e., only 50 cc of water). At all included ECT-sessions, continuous electroencephalogram (EEG) and the clinical reorientation time (ROT) was recorded. Orange color indicates baseline, postictal, or follow-up arterial spin labeling magnetic resonance imaging (ASL-MRI) measurements. Figure taken from Verdijk et al.^15^


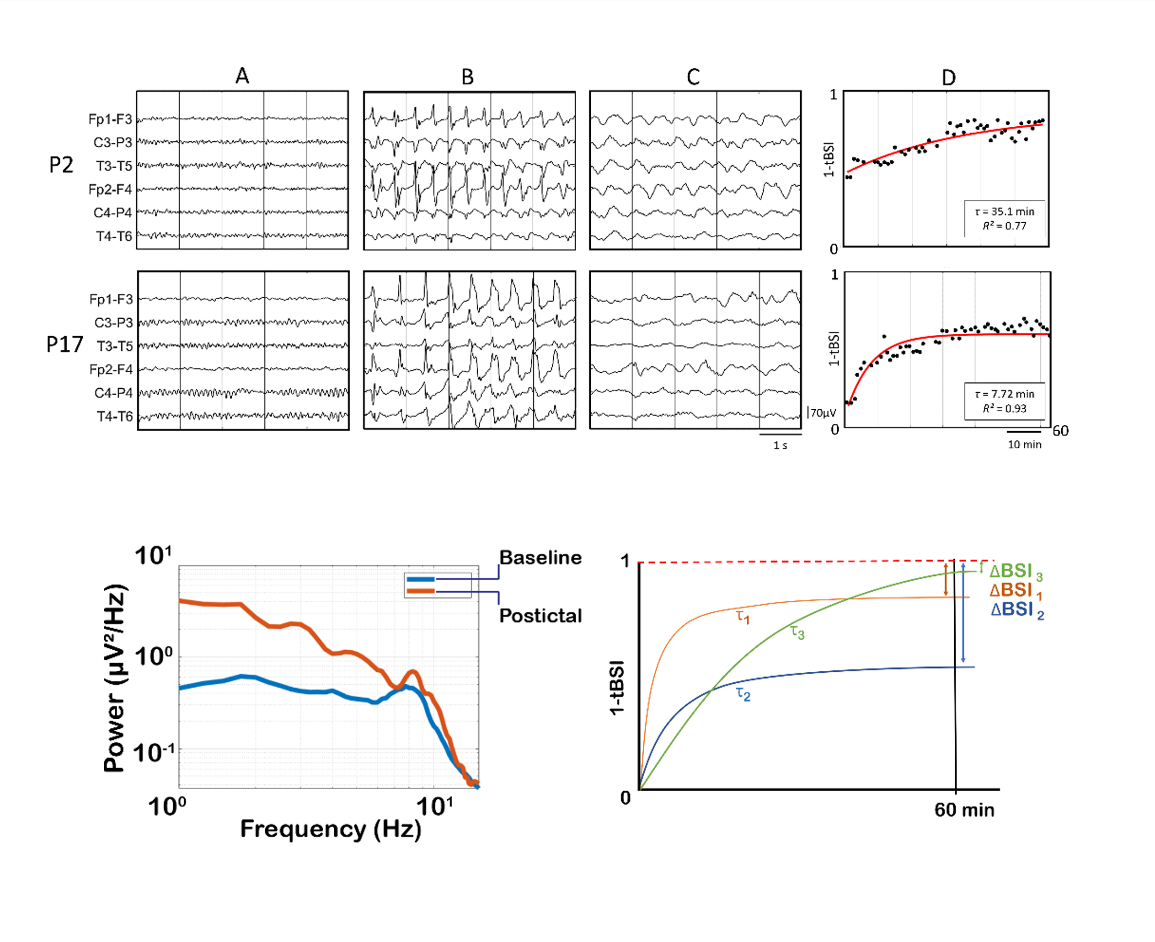


**Figure S2.** **Illustration of the postictal electrophysiological outcome measures**. Top panels show 5 s electroencephalogram (EEG) segments (A: Baseline, B: Seizure, C: Postictal) and postictal EEG recovery (D: temporal brain symmetry index, and left lower panel with power spectral densities of baseline and a postictal segment). Right lower panel: Larger values of τ (τ3 > τ2 > τ1) reflect a slower *speed* of postictal EEG recovery to its stationary value. *Extent* of postictal EEG recovery (ΔBSI) indicates how much the postictal EEG deviates from its previous baseline value at t = 60 min. Figures taken from Pottkämper 2023.^18^


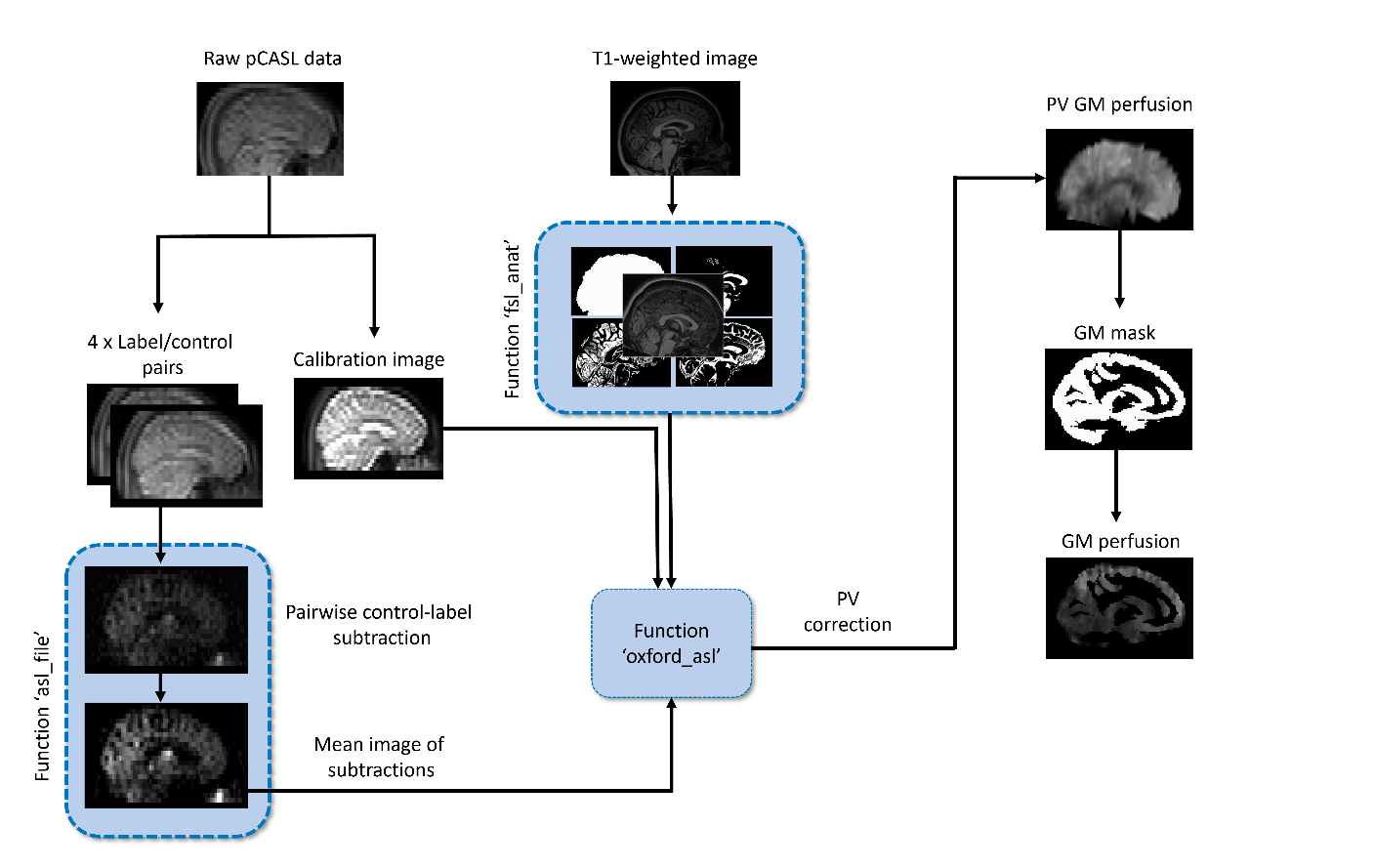


**Figure S3.** **Schematic representation of the preprocessing of arterial spin labelling magnetic resonance imaging (ASL-MRI) and T1W image workflow**. pCASL = pseudo continuous arterial spin labeling; CBF = cerebral blood flow; PV = partial volume; GM = gray matter. Taken from Pottkämper et al. (2022).

**Supplementary Results**

**Table S1. Patient-, electroconvulsive therapy (ECT)-, and trial-characteristics of the SYNAPSE trial**

| ***Characteristics*** | | ***Patient population for EEG analyses***  ***(n=33)*** | ***Patient population for ASL-MRI analyses (n=24)*** |
| --- | --- | --- | --- |
| *Patient characteristics* | | | |
| Age in years, median (IQR; range) | | 53 (21.3; 24 – 82) | 56 (22.5; 24 – 82) |
| Female sex, n (%) | | 19/33 (56) | 15/24 (63) |
| ECT characteristics | | | |
| Bilateral electrode placement at the end of the ECT-course, n (%) | | 24/33 (73) | 15/24 (63) |
| Electrical charges to elicit seizures in milliCoulombs, median (IQR; range) | | 304.7 (228.8; 125.6 – 813.0) | 304.4 (250.2; 125.6 – 813.0) |
| Seizure duration of all included ECT-sessions during the ECT-course in seconds, median (IQR; range) | | 51 (25.5; 16.3 – 178.2) | 55 (24.5; 19.6 – 140.1) |
| Total number of ECT-sessions during the ECT-course, median (IQR; range) | | 12 (9; 7 – 100) | 12 (8; 8 – 100) |
| Concomitant psychopharmacological drug use during the ECT-course, n (%) | |  | |
|  | Antidepressants | 24/33 (73) | 18/24 (75) |
|  | Antipsychotics | 23/33 (70) | 17/24 (71) |
|  | Antiepileptics | 7/33 (21) | 4/24 (17) |
|  | Benzodiazepines | 20/33 (61) | 17/24 (71) |
|  | Lithiumcarbonate | 2/33 (1) | 2/24 (8) |
| Number of patients needing medication for severe postictal symptoms after ECT*, n (%) | | 12/33 (36) | 8/24 (33) |
| *Trial characteristics* | | | |
| Number of EEGs/ASL-MRI scans per intervention, n (%) | |  | |
|  | Acetaminophen | 100/300 (33) | 24/72 (33) |
|  | Nimodipine | 99/300 (33) | 24/72 (33) |
|  | Placebo | 101/300 (34) | 24/72 (33) |
| Interval between administration of study medication and application of ECT-stimulus in minutes, median (IQR; range)** | |  | |
|  | Acetaminophen | 145.5 (31.8; 221 - 50) | 144.5 (27; 212 - 93) |
|  | Nimodipine | 137 (36; 265 - 88) | 136 (24; 265 - 100) |
|  | Placebo | 147 (44; 245 - 77) | 146 (34.5; 245 - 77) |
| Interval between application of the ECT-stimulus and postictal ASL-MRI acquisition in minutes, median (IQR; range) | | NA | 64 (15; 35 – 94) |

ASL = arterial spin labeling; BL = bifrontotemporal; EEG = electroencephalography; ECT = electroconvulsive therapy; IQR = inter quartile range; EEG = electroencephalogram; MRI = magnetic resonance imaging; NA = not applicable

*Postictal medication consisted of a single dose of midazolam, ranging between 2-5 mg.

**Differences in medication administration were tested statistically between the interventions and revealed no significant differences (p=0.482).

**Table S2. Results of Bayesian mixed model analyses according to the statistical analysis plan**

|  | **Speed of postictal EEG recovery (τ, min)** | | | | | | **Equivalence test** | |
| --- | --- | --- | --- | --- | --- | --- | --- | --- |
| *Predictors* | *Estimate* | *CI95 lower* | *CI95 upper* | *Estimate* | *CI95 lower* | *CI95 upper* | *ROPE [-1.4, 1.4]* | |
| Intercept | 10.42 | 2.65 | 40.14 | 10.56 | 2.80 | 39.69 |  |  |
| Acetaminophen versus Placebo | 1.13 | 0.92 | 1.40 |  |  |  | Accepted |  |
| Nimodipine versus Placebo | 1.07 | 0.87 | 1.31 |  |  |  | Accepted |  |
| Time | 1.03 | 1.00 | 1.07 | 1.03 | 1.00 | 1.07 | Accepted | Accepted |
| Electrode placement (Unilateral) | 0.81 | 0.49 | 1.34 | 0.82 | 0.50 | 1.33 | Accepted | Accepted |
| Age (years) | 0.99 | 0.97 | 1.01 | 0.99 | 0.97 | 1.01 | Accepted | Accepted |
| Sex (female) | 0.79 | 0.43 | 1.45 | 0.80 | 0.44 | 1.45 | Accepted | Accepted |
| Treatment versus Placebo |  |  |  | 1.10 | 0.91 | 1.31 |  | Accepted |
|  | **Extent of postictal EEG recovery (ΔBSI)** | | | | | | **Equivalence test** | |
|  | *Estimate* | *CI95 lower* | *CI95 upper* | *Estimate* | *CI95 lower* | *CI95 upper* | *ROPE [-0.01, 0.01]* | |
| Intercept | 0.42 | 0.26 | 0.65 | 0.42 | 0.27 | 0.66 |  |  |
| Acetaminophen versus Placebo | 0.99 | 0.89 | 1.09 |  |  |  | Undecided |  |
| Nimodipine versus Placebo | 0.93 | 0.84 | 1.03 |  |  |  | Undecided |  |
| Time | 0.99 | 0.97 | 1.01 | 0.99 | 0.97 | 1.00 | Undecided | Undecided |
| Electrode placement (Unilateral) | 1.02 | 0.85 | 1.25 | 1.02 | 0.85 | 1.24 | Undecided | Undecided |
| Age (years) | 1.00 | 0.99 | 1.01 | 1.00 | 0.99 | 1.01 | Accepted | Accepted |
| Sex (female) | 0.98 | 0.81 | 1.19 | 0.97 | 0.81 | 1.19 | Undecided | Undecided |
| Treatment versus Placebo |  |  |  | 0.96 | 0.88 | 1.05 |  | Undecided |
|  | **Clinical reorientation time (ROT, min)** | | | | | | **Equivalence test** | |
| *Predictors* | *Estimate* | *CI95 lower* | *CI95 upper* | *Estimate* | *CI95 lower* | *CI95 upper* | *ROPE [-1.9, 1.9]* | |
| Intercept | 0.72 | 0.14 | 3.75 | 0.83 | 0.16 | 4.39 |  |  |
| Acetaminophen versus Placebo | 1.24 | 0.86 | 1.77 |  |  |  | Accepted |  |
| Nimodipine versus Placebo | 1.15 | 0.90 | 1.47 |  |  |  | Accepted |  |
| Time | 0.96 | 0.91 | 1.01 | 0.95 | 0.90 | 1.00 | Accepted | Accepted |
| Electrode placement (Unilateral) | 0.78 | 0.42 | 1.45 | 0.77 | 0.41 | 1.47 | Accepted | Accepted |
| Age (years) | 1.01 | 0.98 | 1.03 | 1.01 | 0.98 | 1.03 | Accepted | Accepted |
| Sex (female) | 0.69 | 0.34 | 1.38 | 0.65 | 0.32 | 1.29 | Accepted | Accepted |
| Treatment versus Placebo |  |  |  | 1.24 | 0.99 | 1.55 |  | Accepted |
|  | **Change in postictal *global* CBF (gCBF, ml/100g/min)** | | | | | | **Equivalence test** | |
|  | *Estimate* | *CI95 lower* | *CI95 upper* |  | | | *ROPE [-1.7, 1.7]* | |
| Intercept | -8.38 | -31.27 | 14.46 |  |  |  |  |  |
| Acetaminophen versus Placebo | 2.47 | -1.36 | 6.24 |  |  |  | Undecided |  |
| Nimodipine versus Placebo | -2.12 | -5.69 | 1.46 |  |  |  | Undecided |  |
| Electrode placement (Unilateral) | -0.64 | -8.92 | 7.60 |  |  |  | Undecided |  |
| Age (years) | -0.01 | -0.30 | 0.29 |  |  |  | Accepted |  |
| Sex (female) | -0.27 | -8.73 | 8.44 |  |  |  | Undecided |  |
| Seizure duration (s) | -0.08 | -0.20 | 0.03 |  |  |  | Accepted |  |
| Δt ASL acquisition (min) | 0.16 | -0.01 | 0.33 |  |  |  | Accepted |  |

Note: Bayesian analyses were performed twice, with the same outcome variables, however, with an additional fixed effect active treatment (i.e., acetaminophen or nimodipine) versus placebo. However, it was not possible to run the analysis on treatment vs placebo for postictal global CBF as both acetaminophen or nimodipine had opposite effects. Fixed effect time refers to the number of the ECT-session. EEG = electroencephalography, CBF = cerebral blood flow, CI = credibility interval, ROPE = regional of practical equivalence, Δt ASL acquisition = time interval between the electroconvulsive therapy stimulus and arterial spin labeling acquisition. Empty cells indicate that fixed effects were not included in the respective model; Accepted = The posterior distribution falls completely within the ROPE, leading to acceptance of the null hypothesis (i.e., there is no effect on the outcome measure); Undecided = The posterior distribution falls partly within the ROPE, which prevents definitive conclusion.


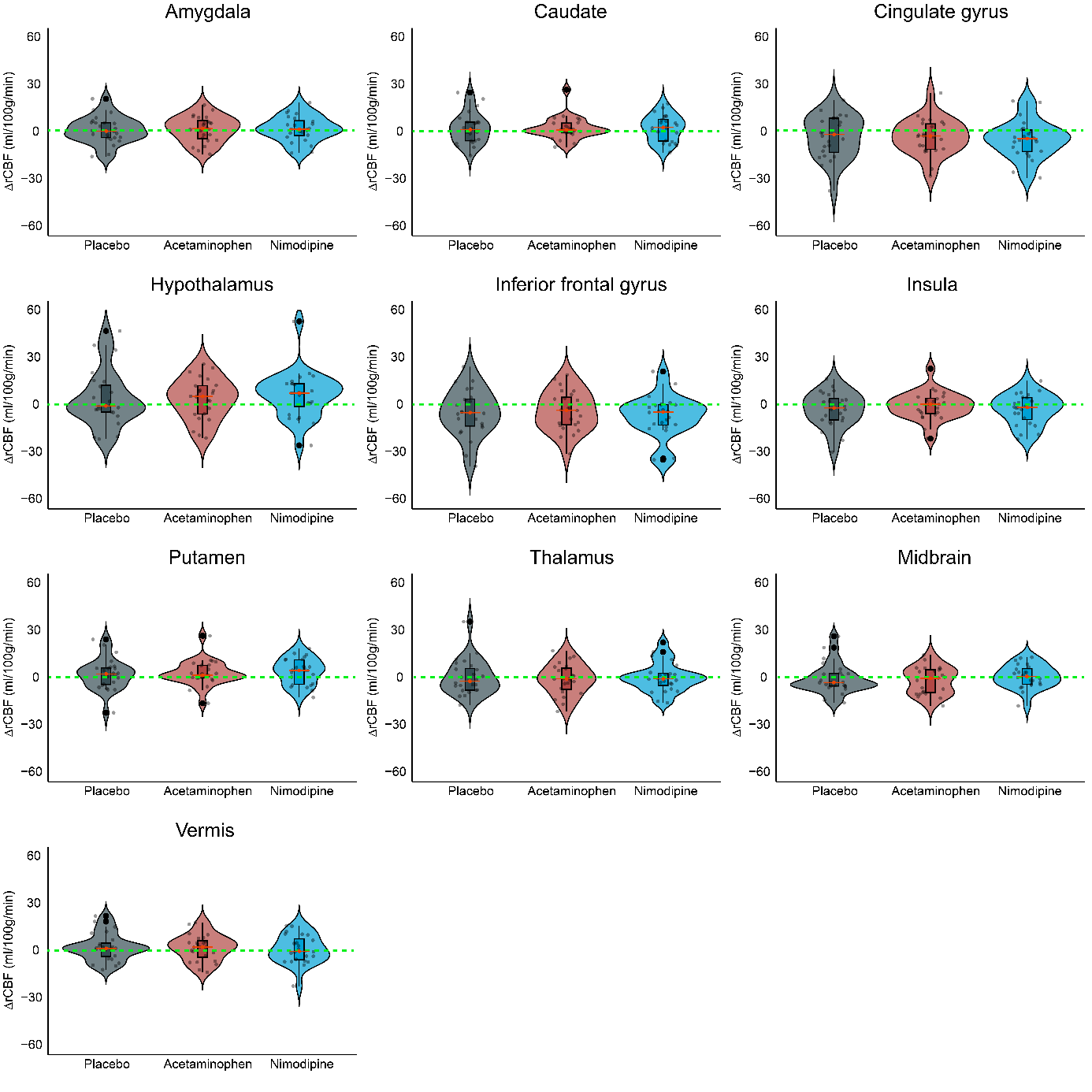


**Figure S4. In all pre-selected regions-of-interest in the postictal state, no effects of treatment with acetaminophen or nimodipine compared to placebo was found on change in regional cerebral blood flow (ΔrCBF).** The dashed green lines indicate no change of postictal CBF with respect to baseline. Red lines indicate median (change in) CBF.

**Table S3**. **Results of Bayesian generalized mixed model analyses regarding perfusion changes in regions-of-interest**

|  | **Change in amygdala CBF (ml/100g/min)** | | **Change in caudate CBF (ml/100g/min)** | | **Change in cingulate gyrus CBF (ml/100g/min)** | | **Change in hypothalamus CBF (ml/100g/min)** | | **Change in inferior frontal gyrus CBF (ml/100g/min)** | | **Change in insula CBF (ml/100g/min)** | | **Change in putamen CBF (ml/100g/min)** | | **Change in thalamus CBF (ml/100g/min)** | | **Change in midbrain CBF (ml/100g/min)** | | **Change in vermis CBF (ml/100g/min)** | |
| --- | --- | --- | --- | --- | --- | --- | --- | --- | --- | --- | --- | --- | --- | --- | --- | --- | --- | --- | --- | --- |
| *Predictors* | *Estimate* | *CI (95%)* | *Estimate* | *CI (95%)* | *Estimate* | *CI (95%)* | *Estimate* | *CI (95%)* | *Estimate* | *CI (95%)* | *Estimate* | *CI (95%)* | *Estimate* | *CI (95%)* | *Estimate* | *CI (95%)* | *Estimate* | *CI (95%)* | *Estimate* | *CI (95%)* |
| Intercept | -15.48 | -31.67 – 0.72 | 12.92 | -4.89 – 30.44 | 0.72 | -48.82 – 49.27 | -6.27 | -41.52 – 29.14 | -23.54 | -75.26 - 28.55 | 2.56 | -17.44 – 22.37 | 13.09 | -3.88 – 30.07 | -5.91 | -48.01 – 36.46 | 23.11 | -11.33 – 57.86 | -7.85 | -42.91 – 27.88 |
| Acetaminophen versus placebo | -0.93 | -3.68 – 1.97 | 2.88 | -0.61 – 6.35 | -0.33 | -4.18 – 3.49 | -3.50 | -12.09 – 4.95 | 1.21 | -2.94 – 5.41 | 3.21 | -0.66 – 7.06 | 0.24 | -3.32 – 3.77 | -1.01 | -4.63 – 2.62 | -1.27 | -4.84 – 2.30 | -0.44 | -4.27 – 3.41 |
| Nimodipine versus placebo | 0.79 | -1.35 – 2.94 | 0.16 | -2.51 – 2.80 | -1.69 | -5.49 – 2.06 | 2.62 | -4.09 – 9.26 | -2.47 | -6.66 – 1.76 | 0.06 | -2.91 – 3.08 | 1.67 | -1.10 – 4.34 | -0.18 | -3.75 – 3.41 | 1.40 | -2.11 – 5.00 | .1.52 | -5.27 – 2.26 |
| Seizure duration (s) | 0.11 | 0.04 – 0.18 | -0.03 | -0.12 – 0.06 | -0.01 | -0.25 – 0.23 | -0.08 | -0.27 – 0.11 | 0.13 | -0.14 – 0.39 | -0.01 | -0.11 – 0.09 | 0.04 | -0.04 – 0.13 | -0.08 | -0.30 – 0.13 | -0.21 | -0.39 - -0.02 | 0.15 | -0.04 – 0.35 |
| Age (years) | 0.13 | -0.09 – 0.36 | 0.08 | -0.15 – 0.31 | 0.10 | -0.43 – 0.64 | -0.06 | -0.44 – 0.33 | 0.04 | -0.52 – 0.60 | -0.11 | -0.37 – 0.15 | -0.20 | -0.41 – 0.01 | 0.04 | -0.41 – 0.49 | -0.10 | -0.44 – 0.24 | -0.00 | -0.36 – 0.35 |
| Sex (female) | -1.53 | -8.42 – 5.35 | 1.03 | -5.69 – 7.87 | -6.29 | -20.40 – 7.87 | -2.87 | -13.48 – 7.70 | 7.33 | -7.54 – 21.79 | 6.22 | -1.34 – 13.86 | -4.93 | -11.06 – 1.13 | 4.98 | -6.87 – 16.55 | -3.24 | -11.69 | -1.97 | -10.89 – 6.89 |
| Electrode placement (Unilateral) | 4.53 | -2.92 – 12.22 | -1.92 | -9.40 – 5.49 | 3.14 | -7.06 – 13.65 | -3.47 | -15.15 – 8.12 | -5.38 | -16.64 – 5.67 | 3.41 | -4.97 – 11.64 | 0.36 | -6.10 – 6.91 | -4.95 | -13.88 – 3.60 | -0.92 | -7.26 – 5.38 | -1.55 | -8.06 – 5.07 |
| Δt ASL acquisition (min) | 0.02 | -0.14 – 0.18 | -0.21 | -0.40– -0.02 | -0.08 | -0.27 – 0.11 | 0.36 | -0.09 – 0.82 | 0.10 | -0.11 – 0.32 | -0.07 | -0.29 – 0.14 | -0.01 | -0.21 – 0.18 | 0.11 | -0.08 – 0.30 | -0.05 | -0.22 – 0.11 | 0.06 | -0.12 – 0.24 |
| ROPE interpretation acetaminophen versus placebo | Undecided | | Undecided | | Undecided | | Undecided | | Undecided | | Undecided | | Undecided | | Undecided | | Undecided | | Undecided | |
| ROPE interpretation nimodipine versus placebo | Undecided | | Undecided | | Undecided | | Undecided | | Undecided | | Undecided | | Undecided | | Undecided | | Undecided | | Undecided | |
| ROPE interpretation seizure duration | Undecided | | Undecided | | Undecided | | Undecided | | Undecided | | Undecided | | Undecided | | Undecided | | Undecided | | Undecided | |
| ROPE interpretation Δt ASL acquisition | Undecided | | Undecided | | Undecided | | Undecided | | Undecided | | Undecided | | Undecided | | Undecided | | Undecided | | Undecided | |

Note: Δt ASL acquisition = time interval between the electroconvulsive therapy stimulus and arterial spin labeling acquisition; CI = credibility interval; ROPE = region of practical equivalence; CBF = cerebral blood flow

**Table S4. Voxel-wise results comparing nimodipine with placebo and acetaminophen treatment showing decreased postictal cerebral blood flow with nimodipine treatment compared to placebo or acetaminophen treatment.**

|  | **Anatomical location based on Talairach atlas** | **Voxel cluster size** | ***p*-value** | ***T*-value** | **MNI coordinates (x, y, z)** | | |
| --- | --- | --- | --- | --- | --- | --- | --- |
| Omnibus F-test | Left precuneus | 86 | 0.030 | 12.91 | -8 | -64 | 58 |
|  | Right superior parietal lobule | 100 | 0.014 | 11.27 | 18 | -62 | 60 |
| Post-hoc comparisons | | | | | | | |
| Nimodipine versus placebo | Left precuneus | 212 | 0.001 | 4.55 | -10 | -54 | 54 |
| Nimodipine versus Acetaminophen | Right superior parietal lobule | 781 | 0.000 | 4.93 | 26 | -78 | 50 |
|  | Left precuneus | 243 | 0.000 | 4.81 | -8 | -62 | 64 |
|  | Left superior parietal lobule | 169 | 0.004 | 4.27 | -28 | -64 | 50 |

**Table S5.** **Results of Bayesian mixed model analyses regarding *global* and *regional* perfusion changes in regions-of-interest and clinical reorientation time (ROT)**

|  | **Change in global CBF (ml/100g/min)** | | **Change in amygdala CBF (ml/100g/min)** | | **Change in caudate CBF (ml/100g/min)** | | **Change in cingulate gyrus CBF (ml/100g/min)** | | **Change in hypothalamus CBF (ml/100g/min)** | | **Change in inferior frontal gyrus CBF (ml/100g/min)** | | **Change in insula CBF (ml/100g/min)** | | **Change in putamen CBF (ml/100g/min)** | | **Change in thalamus CBF (ml/100g/min)** | | **Change in midbrain CBF (ml/100g/min)** | | **Change in vermis CBF [ml/100g/min]** | |
| --- | --- | --- | --- | --- | --- | --- | --- | --- | --- | --- | --- | --- | --- | --- | --- | --- | --- | --- | --- | --- | --- | --- |
| *Predictors* | *Estimate* | *CI (95%)* | *Estimate* | *CI (95%)* | *Estimate* | *CI (95%)* | *Estimate* | *CI (95%)* | *Estimate* | *CI (95%)* | *Estimate* | *CI (95%)* | *Estimate* | *CI (95%)* | *Estimate* | *CI (95%)* | *Estimate* | *CI (95%)* | *Estimate* | *CI (95%)* | *Estimate* | *CI (95%)* |
| Intercept | 0.10 | -5.06 – 5.33 | 2.06 | -1.85 – 6.02 | 2.30 | -2.03 – 6.57 | 0.26 | -6.56 – 7.26 | 8.80 | 0.50 – 17.11 | -6.78 | -14.45 – 0.91 | -3.49 | -8.51 – 1.48 | 2.49 | -1.83 – 6.85 | 0.74 | -5.13 – 6.48 | -1.22 | -6.48 – 3.73 | 0.26 | -4.82 – 5.33 |
| ROT | -0.08 | -0.17 – 0.01 | -0.01 | -0.09 – 0.06 | 0.00 | -0.09 – 0.09 | -0.07 | -0.18 – 0.03 | -0.12 | -0.31 – 0.08 | 0.03 | -0.08 – 0.14 | 0.04 | -0.05 – 0.14 | 0.02 | -0.07 – 0.11 | -0.02 | -0.11 – 0.07 | 0.02 | -0.07 – 0.10 | 0.03 | -0.05 – 0.11 |
| ROPE interpretation ROT | Undecided | | Undecided | | Undecided | | Undecided | | Undecided | | Undecided | | Undecided | | Undecided | | Undecided | | Undecided | | Undecided | |

Note: ROT = reorientation time, CI = credibility interval, CBF = cerebral blood flow, ROPE = region of practical equivalence


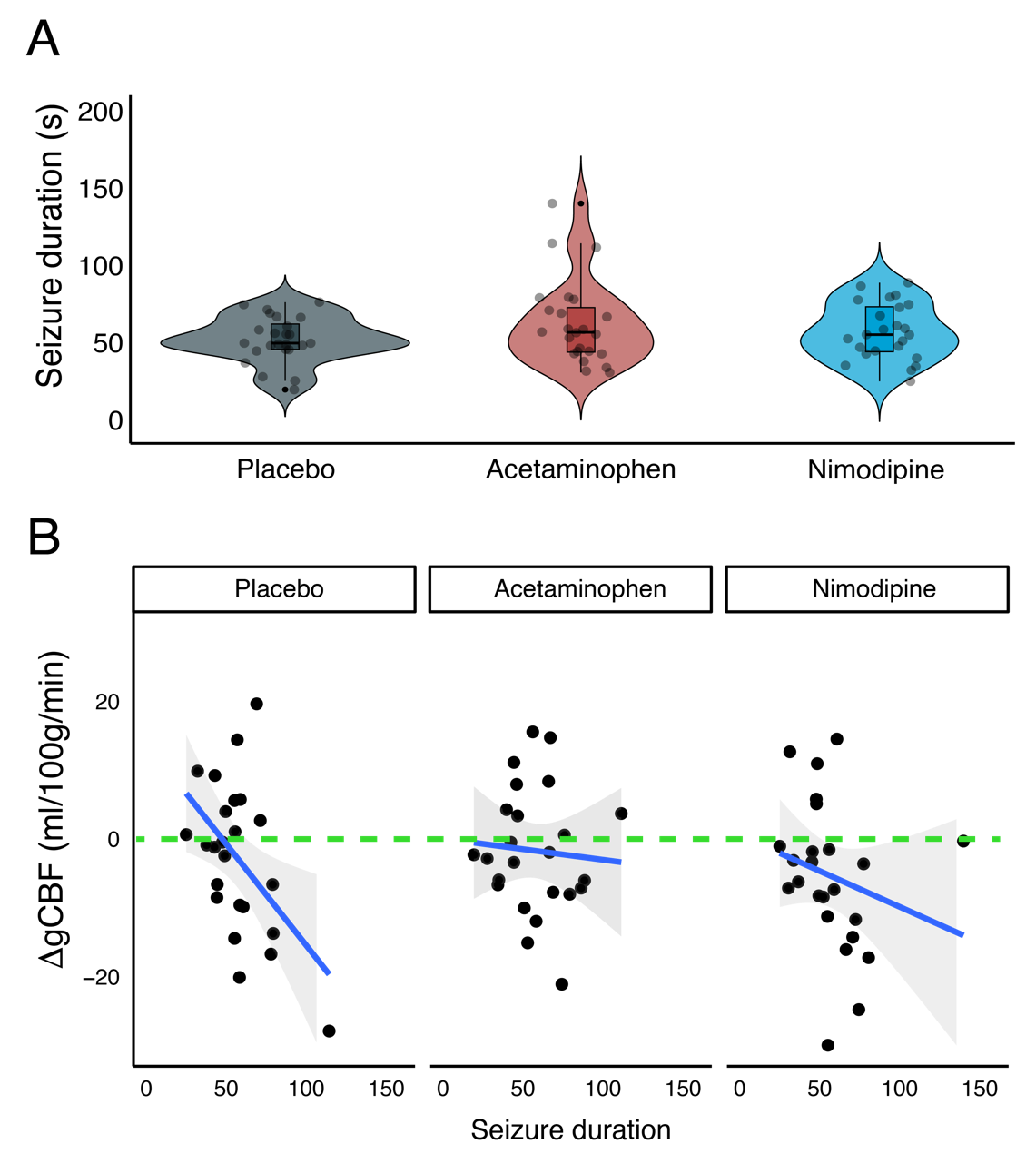


**Figure S5.** Distribution of seizure duration after pre-treatment with placebo, acetaminophen, or nimodipine (A) and association between seizure duration and ΔgCBF after pre-treatment with acetaminophen, nimodipine, or placebo (B). No significant overall effect (intervention or seizure duration) or interaction effect (intervention * seizure duration) were detected. However, visually, it seems that when patients received acetaminophen, there was less postictal decrease of gCBF. Only data from patients with MRI-scans are provided (N = 24 patients, N = 72 data points).

**Table S6.** **Results of Bayesian mixed model analysis investigating the interaction effect of seizure duration and the intervention on postictal global cerebral blood flow changes**

|  | **ΔCBF (ml/100g/min)** | |
| --- | --- | --- |
| *Predictors* | *Estimates* | *CI (95%)* |
| Intercept | 5.25 | -17.61 – 27.45 |
| Seizure duration (s) | -0.13 | -0.32 – 0.07 |
| Acetaminophen versus placebo | -5.08 | -17.41 – 7.11 |
| Nimodipine versus placebo | -5.61 | -18.67 – 7.11 |
| Seks (female) | -1.59 | -10.53 – 7.12 |
| Age (years) | 0.00 | -0.28 – 0.30 |
| Interaction seizure duration * Acetaminophen versus placebo | 0.11 | -0.10 – 0.32 |
| Interaction seizure duration * Nimodipine versus placebo | 0.06 | -0.15 – 0.27 |


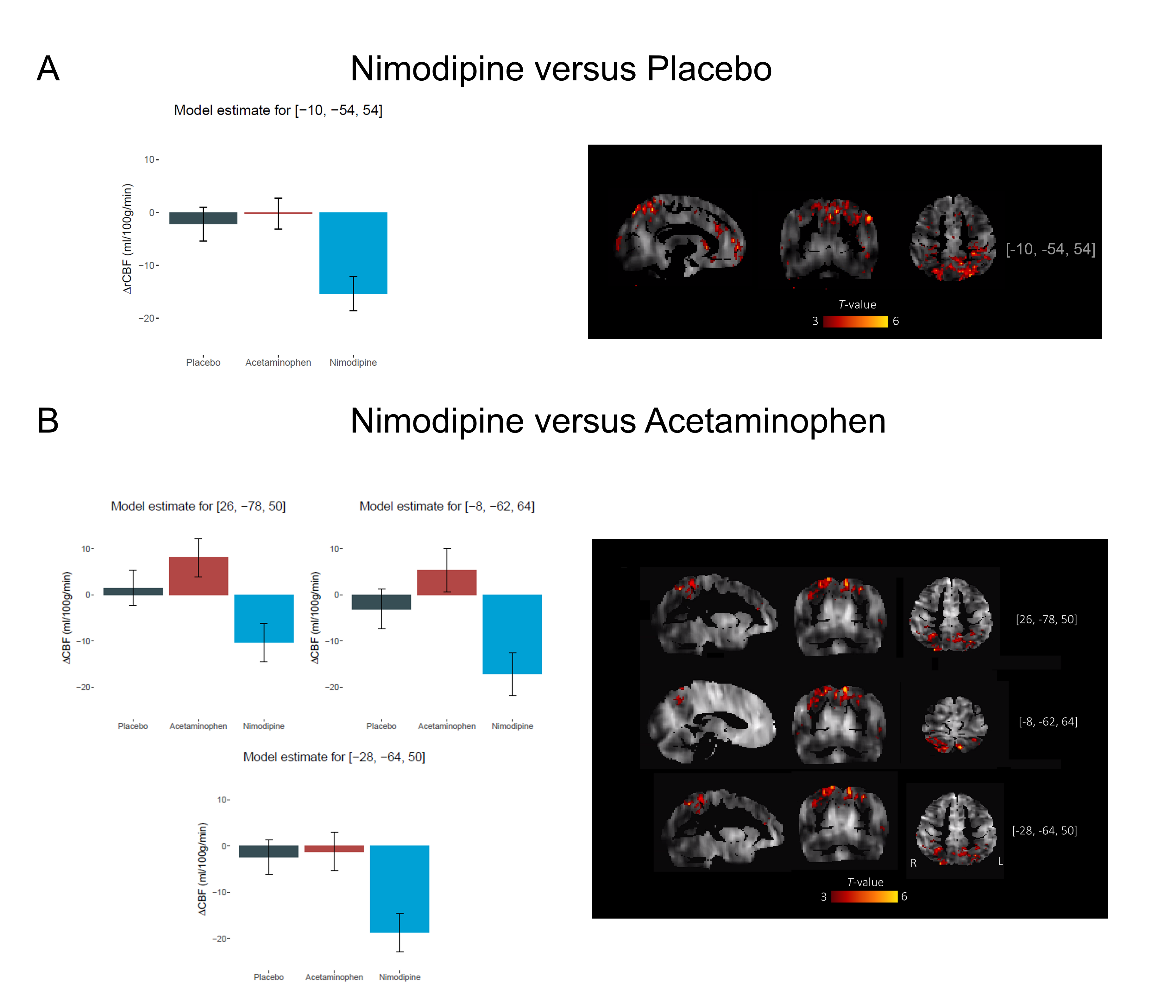


**Figure S6. Results of voxel-wise CBF analyses.** A) Voxel-wise comparison between nimodipine and placebo treatment, indicating a significant cluster in the precuneus shown in CBF maps on the right with corresponding model Estimate on the left. B) Voxel-wise comparison between nimodipine and acetaminophen treatment, highlighting three significant clusters, one in the precuneus, and two in the superior parietal lobule. Anatomical locations are defined based on the Tailairach Daemon atlas. The *T*-value map is superimposed to a patient’s ΔCBF map, with *T*-values ranging between 3 and 6. Clusters are shown with *p* < 0.05 (Family-wise error corrected). Bar plots with model Estimate are shown with standard errors for visual representation. CBF = cerebral blood flow
